# Supplementary material for: Resolving the full spectrum of human genome variation using Linked-Reads
Source: Genome Res. 2019 Apr;29(4):635–45. doi: 10.1101/gr.234443.118 (PMC6442396; doi:10.1101/gr.234443.118)
Supplement: Supplemental Material [file supp_gr.234443.118_Supplemental_longranger-2.2.zip › longranger-2.2.tar.bz2!/lr2.2-public/lib/stan/cmdstan/stan/doc/api/html/agrad_2fwd_2matrix_2rows__dot__self_8hpp_source.html]

Stan: src/stan/agrad/fwd/matrix/rows\_dot\_self.hpp Source File


|  |  |
| --- | --- |
|  | Stan  2.5.0  probability, sampling & optimization |


- Main Page
- Related Pages
- Namespaces
- Classes
- Files

- File List
- File Members

All Classes Namespaces Files Functions Variables Typedefs Enumerations Enumerator Friends Macros Pages

- src
- stan
- agrad
- fwd
- matrix

rows\_dot\_self.hpp

Go to the documentation of this file.

1 #ifndef STAN\_\_AGRAD\_\_FWD\_\_MATRIX\_\_ROWS\_DOT\_SELF\_HPP

2 #define STAN\_\_AGRAD\_\_FWD\_\_MATRIX\_\_ROWS\_DOT\_SELF\_HPP

3

4 #include <vector>

5 #include <stan/math/matrix/Eigen.hpp>

6 #include <stan/math/matrix/typedefs.hpp>

7 #include <stan/agrad/fwd/fvar.hpp>

8 #include <stan/agrad/fwd/matrix/dot\_self.hpp>

9

10 namespace stan {

11  namespace agrad {

12

13  template<typename T, int R,int C>

14  inline Eigen::Matrix<fvar<T>,R,1>

15  rows\_dot\_self(const Eigen::Matrix<fvar<T>,R,C>& x) {

16  Eigen::Matrix<fvar<T>,R,1> ret(x.rows(),1);

17  for (size\_type i = 0; i < x.rows(); i++) {

18  Eigen::Matrix<fvar<T>,1,C> crow = x.row(i);

19  ret(i,0) = dot\_self(crow);

20  }

21  return ret;

22  }

23  }

24 }

25 #endif

stan::agrad::dot\_self

fvar< T > dot\_self(const Eigen::Matrix< fvar< T >, R, C > &v)

**Definition:** dot\_self.hpp:16

typedefs.hpp

stan::agrad::fvar

**Definition:** fvar.hpp:13

dot\_self.hpp

stan::agrad::size\_type

Eigen::Matrix< double, Eigen::Dynamic, Eigen::Dynamic >::Index size\_type

**Definition:** typedefs.hpp:14

fvar.hpp

stan::agrad::rows\_dot\_self

Eigen::Matrix< fvar< T >, R, 1 > rows\_dot\_self(const Eigen::Matrix< fvar< T >, R, C > &x)

**Definition:** rows\_dot\_self.hpp:15

Eigen.hpp


---

[ Stan Home Page ]

*© 2011–2014,
Stan Development Team.*
